# Evaluating the effectiveness of physical exercise in improving standardized testing performances through attention indices

**DOI:** 10.1002/brb3.2800

**Published:** 2022-10-31

**Authors:** Jeffrey Huang, Hubert Huang, Benjamin Chang, Jocelyn Ho

**Affiliations:** ^1^ Department of Science Pacific American School Hsinchu Taiwan; ^2^ Whiting School of Engineering Johns Hopkins University Baltimore Maryland USA; ^3^ School of Computer Science Georgia Institute of Technology Atlanta Georgia USA

**Keywords:** attention, digital image processing, eye movement, physical exercise, reading speed, standardized tests

## Abstract

**Introduction:**

In the United States, standardized tests have risen in prevalence, extending their importance from education placement to employment. Attention is crucial to improving testing performance. Past studies have established that acute, coordinative, aerobic exercise improves attention, which is measured by the D2 Attention Test, emotional analysis, reading time, and eye movement tracking. No studies have drawn connections between physical exercise's quantifiable improvement in attention to improvements in standardized tests; therefore, this study would attempt to do so.

**Methods:**

This study defines attention to be positively related to reading speed and negatively related to the number of eye drifts. High school students were selected to read and answer two reading passages from an SAT (Scholastic Assessment Test) exam, before and after a short 80% intensity run. Their reading times, facial video, and test scores were recorded. Dlib plots the facial landmark and OpenCV tracks movement of the pupil.

**Results:**

Through paired‐samples *t*‐tests, this study found out that after exercise, subjects displayed increased reading speed and fewer eye drifts, coupled with increased mean scores.

**Conclusion:**

Thus, this study demonstrated that running, as an acute, coordinative, aerobic exercise, helps increase the testing performance of the SAT reading section by measuring attention. Future research could focus on including head movement as an attention index, replicate the experiment on different standardized tests or exercises, and conduct natural experiments to better simulate real‐life conditions to increase applicability.

## Graphical Abstract

Paired‐samples t‐tests showed that after exercise, subjects displayed increased reading speed and fewer eye drifts, coupled with increased mean scores. This study demonstrated that running, an acute, coordinative, aerobic exercise, improves attention and increases the testing performance of the SAT reading.

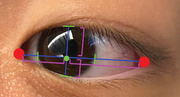

## 1 INTRODUCTION

Since the 20th century, standardized testing has become an important aspect of the United States’ education system (Himelfarb, 2019). In response to the country's poor educational outcome among other developed countries, which have greater oversight over testing and curriculum, Congress passed the No Child Left Behind Act in an attempt to regularize education (Benjamin & Pashler, 2015). Specifically, standardized tests, indicators of student academic performance and aptitude, became more widespread to assess the quality of education (Wiliam, 2010). A study by the Council of the Great City Schools indicated that in the country's largest urban districts, students take an average of 112 standardized tests from pre‐kindergarten to grade 12 (Hart et al., 2015). Recently, the increasing number of college applicants increased colleges’ reliance on standardized tests to tighten admission under limited resources (Zwick, 2017). The impact of standardized tests goes beyond college admissions: “gatekeepers of America's meritocracy—educators, academic institutions, and employers—have used test scores to label people as bright or not bright, as worthy academically or not worthy” (Sacks, 2000). Regardless of the tests’ fairness, they impact students’ chances of success.

However, some critics argue that standardized tests are unrepresentative of students’ capabilities (Kempf, 2016). In addition, occasional fluctuations in testing performance could overshadow the cumulative performance of students in class (Wingate & Tomes, 2017). Sievertsen et al. (2016) affirm this statement, arguing that cognitive fatigue can induce a decrease in attentiveness, which causes significant drops in standardized testing performance. Thus, attention can play an essential role in the improvement of standardized testing.

In light of the growing importan ce of standardized tests, past studies have found physical exercise as a potential method of improving testing performance. Coe et al. (2006) observed that vigorous physical exercise increases academic achievement, which was measured by a national standardized test. Although this correlation has been established, there is a current paucity of scientific research investigating and quantifying attention in explaining how physical exercise leads to improvements in testing performance.

The SAT (Scholastic Assessment Test), a standardized test for reading, writing, and math, has become widespread in U.S. high school education (Zwick, 2004). Recently, the College Board has announced the test's digital transition by spring of 2024 (College Board, 2021). This study will evaluate the role of attention between physical exercise and standardized tests through the SAT reading test in a digital setting.

## 2 LITERATURE REVIEW

### 2.1 Physical exercise and attention

To understand how physical exercise improves standardized test scores through the enhancement of attention, it is important to look at the existing body of research on the subject. There has been abundant research suggesting that physical exercise enhances student engagement and attention. Spitzer and Hollmann (2013) demonstrated that attention performance increased significantly after physical exercise. Attention was measured through the D2 Attention Test, where subjects identify a certain symbol among other similar symbols. The test measures selective and sustained attention as well as visual scanning speed (Brickenkamp, 1962). Through the D2 Attention Test, Budde et al. (2008) found that acute, coordinative exercise outperformed other types of exercise in improving attention. In a systematic review, de Sousa et al. (2018) found that aerobic exercises were also effective in improving attention. Both de Sousa et al. and Spitzer and Hollmann explained that physical exercise activates the prefrontal cortex, responsible for cognitive and learning abilities. Specifically, the prefrontal cortex allows individuals to stay on task and retain information (Diamond, 2011). Meanwhile, Budde et al. reasoned that acute, coordinative exercise activates the cerebellum, which controls both motor functions and neurobehavioral systems such as attention and working memory. Similarly, Planinsec (2002) argued that cognitive abilities relate most to exercises through coordination and speed of movement. Together, these studies suggested that acute, coordinative, aerobic exercises improve attention.

### 2.2 Attention and testing performance

Previous literature has established the positive relationship between attention and testing performance. Although the sample is limited to children with autism, Koegel et al. (1997) have shown that improving attention and motivation considerably improved standardized testing performance. McConaughy et al. (2009) reported that children with attention deficit hyperactivity disorder scored significantly lower on the Wechsler Individual Achievement Tests due to their lack of ability to concentrate. Similarly, Sievertsen et al. (2016) found that cognitive fatigue induces a lack of attention and decreases standardized testing performance. These two studies show that a lack of attention decreases standardized test scores. It could be inferred that attention is an integral factor in standardized testing performance.

### 2.3 Indices of attention

Although attention has been measured in various contexts, Sinatra et al. (2015) raised concerns about the generalization of attention measurements and argued that the instrumentation of attention must be specified to fit the context of the research question and experimental design. Most of the past literature evaluating the effect of physical exercise on attention utilized the D2 Attention Test to assess attention (de Sousa et al., 2018). Both Spitzer and Hollmann (2013) and Budde et al. (2008) found a positive correlation between the presence of physical exercise and D2 Attention Test scores, but the context of attention was not specified, thus the method of attention measurement was not modified. In the context of this study, which uses standardized reading tests, subjects will simulate the SAT Reading Test through timed reading (College Board, 2022). Although the D2 Attention Test is effective in evaluating visual speed and accuracy, it only pertains to visual scanning and differs from the line‐by‐line reading in standardized reading tests (Katz & Carlisle, 2009; Steinborn et al., 2018). In addition, the results of the D2 Attention Test are more “influenced by a skipping strategy than is the concentration performance measure” (Bates & Lemay, 2004, p. 398).

Unlike the research from Spitzer and Hollmann, Budde et al., and de Sousa et al., who studied the effect of physical exercise on attention, some studies focused on how attention is tested. Instead of using the conventional D2 Attention Test, these studies attempted to devise specific indices, such as eye movements, emotional analysis, and reading times, to better gauge attention based on different contexts. Under the context of self‐paced reading, Miller (2015) defined attention on reading time and eye movements and provided underlying assumptions. He first established attention as a baseline of comprehension, which affects standardized reading texts, because subjects must concentrate on a piece of text before they could cognitively engage with the text. Miller suggested that attention is the quantity and quality of mental resources dedicated to an object as well as the accompanying behaviors. In eye movement, saccades, or rapid movements, and fixations, or pauses at a particular point, determine attention allocation (Duchowski, 2017). Per Miller, a machine tracks the location of the eye and a computer uses the screen layout to trace the part of the screen that is within the fovea. Morimoto and Mimica (2005) tracked the pupil coordinates for more accurate eye movement tracking. In addition, Miller argued that in the case of self‐paced reading, the reading time is positively correlated to attention since subjects fixate on words longer due to the more mental resource devoted to the text. Reading times that are significantly lower than average could indicate that readers skip words or do not fully comprehend. Miller also explained that eye movement tracking could identify words readers skipped or missed, indicating less or a lack of attention dedicated to that portion of the text. Although reading time could be useful in this study, it should be noted that self‐paced reading is different from the SAT reading tests, where time is limited.

Azevedo (2015) synthesized from past literature and provided various methods to measure attention, including emotional analysis, feature selection, and eye tracking. The study recommended the triangulation of other sensors and indices for better detection of student engagement and attention. Aslan et al. (2014) used feature selection, including gaze, body posture, and facial points to create a student engagement classification with accuracies up to 85%–95%. However, the study only specified that the students were in a learning environment and failed to address the specific educational context where this model is beneficial. Leelavathy et al. (2020) proposed the use of a built‐in laptop webcam to deliver real‐time information regarding student engagement in digital learning through emotional analysis and eye movement tracking. After recording, the video was broken down into images and processed through the Haar Cascade Algorithm, which uses edge or line detection to recognize facial features (Viola & Jones, 2001). Additional emotional analysis was added, to match emotion states such as neutral, happy, sad, anger, and surprise to the content of online learning.

Aslan et al. and Leelavathy et al.’s approaches were similar to that of Miller as they did not merely cover the surface‐level visual scanning in the D2 Attention Test. In addition, eye movement was accompanied by facial analysis to provide additional details on the focus of attention. Eye movement tracking fits the context of the SAT reading test, where subjects will read line‐by‐line and produce discernible eye patterns. Although emotional analysis can gauge attention under certain conditions, emotions may be difficult to gauge under standardized testing, which is informative. Furthermore, a lack of attention could mean that participants do not absorb the information, making emotional analysis unfit (Schindler & Bublatzky, 2020).

### 2.4 Gap

Research methods from the aforementioned studies provide an evaluation of attention in the context of reading, learning, and test taking. Most of these indices are based on digital image processing, the way of processing and analyzing digital images through computer algorithms and machine learning, to predict and detect the levels of attention (Reddy, 2018). The D2 Attention Test provides little useful information due to its lack of pattern in visual scanning. Emotional analysis is difficult to gauge student attention because standardized tests do not provoke significant emotion and whether students exhibit specific emotions depends on whether students are attentive in the first place. Indices such as eye‐movement tracking and reading times provide an appropriate means to gauge attention in reading standardized tests.

Coe et al. (2006) suggested the potential of attention as a link between physical exercise and increased standardized testing performance. Although there has been extensive research on attention indices, there is insufficient research applying them in gauging the effect of physical exercise on attention in standardized testing contexts. This study will attempt to measure attention based on reading time and eye movements. Furthermore, it would evaluate and validate the potential of physical exercise in maximizing standardized testing performances, which is in line with past literature.

### 2.5 Hypothesis

This study hypothesizes that the indices of attention, including reading time and eye movements, correlate with performance in standardized tests, which, inferred from past literature, could be improved with acute, coordinative, aerobic exercises.

## 3 METHOD

### 3.1 Overview

This experiment aimed to show the relationship between physical exercise and improved standardized test performance through enhancing attention. Previous research established that physical exercise improves attention, but lacks specificity in terms of attention. Therefore, to contextualize the attention used in test taking, indices such as eye‐movement tracking and reading time were implemented to accurately gauge student attention, which would then be matched with their test scores. A quantitative research method was implemented to numerically determine the difference in attention and test performance of the test before and after physical exercise.

### 3.2 Subject selection

The subjects were selected from high school students in a competitive international high school in Taiwan, where both standardized testing and sports are emphasized. To ensure all subjects have similar effects to physical exercise, they were required to be enrolled in an ongoing sports team, club, or class on a weekly basis. All subjects had given consent to being recorded throughout the experiment, and there were no external incentives in participation to prevent data contamination. Furthermore, this research, which includes collecting human data, has been approved by the Pacific American School High School Institutional Review Board. Before the test, they were given clear instructions on test taking. To minimize the Hawthorne effect, subjects were informed about the intentions of evaluating attention after the experiment (Sedgwick & Greenwood, 2015). After the test, subjects were asked if they have prior knowledge of the passage. If so, the data of the subject would be removed from the study.

### 3.3 Standardized testing

The SAT was selected as the standardized test due to its prevalence in high school (Zwick, 2004). The reading section was selected as the line‐by‐line reading of sentences in paragraphs produces the most discernible pattern to analyze eye movement. Two reading passages were selected from the reading sections of two different Official SAT Practice Tests. Both tests are the first, or literature, passage of the reading section to ensure the similarity of content. The two passages each have 10 different corresponding multiple‐choice questions from the practice test. A laptop was used to project the passage and questions, and the answers were marked by the subjects on a separate bubble sheet. Throughout the experiment, the two passages and questions and their orders taken by the subjects were kept the same. The subjects were given a total of 13 min to read and answer the questions since there are five passages in the SAT reading portion to complete in 65 min (College Board, 2020). To ensure that the two passages had similar difficulty, a controlled experiment required subjects to read the passage and answer the questions for both tests. Their scores, the number of correctly answered questions for each passage, were recorded. After the controlled experiment, in the variable experiment, a separate set of subjects were instructed to read and complete the questions for each passage, one before and one after physical exercise. The subjects were told to read the entire passage first before answering the questions to prevent saccades caused by eye movement from the questions back to the passage, which could be interpreted as signs of distraction and shifted attention from the passage (Jonikaitis et al., 2013). The built‐in webcam from the laptop and a stopwatch recorded the subjects’ face and reading time, respectively, as they read the passage in both tests.

### 3.4 Physical exercise

Physical exercise was the independent variable. Subjects were required to read and complete the questions from two passages, one before physical exercise and one after. Literature established coordinative, acute, and aerobic exercises bring cognitive enhancements, particularly attention (Budde et al., 2008; de Sousa et al., 2018). To maximize the effect of physical exercise on attention, running was selected as an aerobic exercise that requires enough speed and coordination of movement to balance at high, intense speeds (Siddiqui et al., 2010). Before the first test, subjects’ resting heart rates were recorded. Immediately after the first test, subjects were required to conduct exercise for 10 min to be acute as opposed to a long period (Slutsky‐Ganesh et al., 2020). Subjects’ heart rates were immediately recorded after the exercise. A 5‐min recovery was conducted before the second passage (Cantrelle et al., 2020). Throughout this process, subjects would be supervised by the school physical education teacher to reduce the risk of sports injuries.

Exercise intensity was standardized to ensure similar effects of exercise on subjects. Similar studies on the effect of acute treadmill walking on cognition aimed for 60% intensity (Hillman et al., 2009). The desired intensity for this study is 80%, as suggested by Wang et al. (2013), due to the intensive nature of running. Assessments of heart rate before and after exercise have been recommended as a method for establishing exercise intensity (Liguori et al., 2022). Target heart rate (HR_target_) was calculated through the equation Karvonen and Vuorimaa (1988) established, using maximum heart rate (HR_max_), resting heart rate (HR_rest_), and intensity (% intensity).

(1) $$HR_{\mathrm{target}}=\left(HR_{\max}-HR_{\mathrm{rest}}\right)\times \%\;\mathrm{intensity}+HR_{\mathrm{rest}}.$$

Choi et al. (2015) used the following equation to determine the maximum heart rate.

(2) $$HR_{\max}=220-\mathrm{age}.$$

All subjects’ heart rates after exercise passed the target heart rate.

### 3.5 Measuring attention

A stopwatch recorded the reading time of the passage and kept track of the time left for the subjects to complete the test. Due to the word count difference between the two passages, reading speed was calculated by dividing the number of words by the number of seconds to complete the reading for each passage. Although past research assumed that longer reading time indicates enhanced attention, in the context of standardized tests, which require subjects to efficiently read and absorb information, this experiment evaluated reading speeds such that higher reading speeds indicate attentiveness (Miller, 2015; Coltheart, 1987).

**FIGURE 1:**
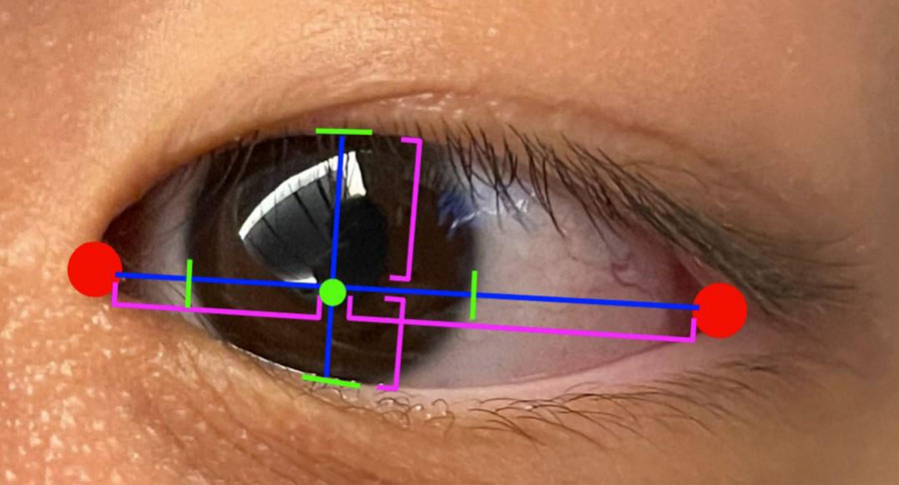
Diagram of pupil position relative to eye

After the two tests, subjects were instructed to scan from the leftmost to the rightmost and topmost to the bottommost edge of the screen, with the camera recording their eye movements. This baseline eye movement would then be compared to subjects’ eye movements during the reading time (Ellis et al., 2011). The previously recorded videos of subjects’ face during the tests were then analyzed. Python programming language was used to conduct digital image processing. The dlib and OpenCV packages were then installed. Dlib detected the frontal face and plotted the coordinates of facial landmarks (Boyko et al., 2018). A pretrained network from Kazemi and Sullivan (2014) was used to detect facial landmarks. Figure 1 shows the diagram of the pupil position relative to the eye. The red two points on the corners of the eye were connected to form a blue, horizontal segment in Figure 1. OpenCV then tracked the pupil position to determine shifts in eye focus (Shang et al., 2019). From OpenCV, the edge of the iris, shown as green segments in Figure 1, could be determined due to the difference in color between the white sclera and the colored iris (Schwarz et al., 2012). The two points on the horizontal segment that intersects the edges of the pupil were marked. The midpoint between these two points, shown as a green dot in Figure 1, produced the horizontal pupil position. A segment perpendicular to the horizontal segment was drawn through the horizontal midpoint. OpenCV was used to differentiate between the iris and the eyelid at the two ends of the vertical segment using color difference. The point of intersection between the vertical and horizontal segment produced two vertical and horizontal subsegments, shown as pink brackets in Figure 1. The ratios of the vertical and horizontal subsegments were calculated. The number of times the horizontal or vertical ratio exceeds the maximum ratios of the baseline eye movements after the two tests was recorded. This number was interpreted as the number of times the eye gaze drifts out of the frame of the laptop, which signifies the shift of attention away from the passage (Zhao et al., 2012).

### 3.6 Data analysis

Paired‐samples *t*‐tests were used to compare the scores, reading speeds, and eye movements, as this test compares the means of two measurements from the same individual taken at two different times (Student, 1908). Levene's test was used to ensure equal variance among samples, which is a precondition of the paired‐samples *t*‐test (Mandelbrot, 1961). IBM SPSS Statistics was used to conduct these tests.

## 4 RESULTS

Table 1 shows the demographic characteristics of the participants. A total of 60 high school students in the international school participated in the research: 30 for the controlled experiment and 30 for the variable experiment with exercise. Since both experiments had samples greater or equal to 30, according to the Central Limit Theorem, the sampling distribution could be approximated to a standard normal distribution (Kwak & Kim, 2017). All of the subjects have signed consent forms. Throughout the experiment, they were supervised by a certified PE teacher. The subjects were all involved in sports teams, clubs, or classes on a weekly basis. Simple random sampling was used within that population; observation of individual participants was independent of that of other participants. There were approximately equal numbers of participants from both genders and age groups.

**TABLE 1:** Demographic characteristic of participants

Characteristics	Control group		Experimental group		Full sample	
	n	%	N	%	n	%
Gender						
Male	15	50	14	46.67	29	48.33
Female	15	50	16	53.33	31	51.67
Age						
16 years	11	36.67	10	33.33	21	35
17 years	10	33.33	10	33.33	20	33.33
18 years	9	30	10	33.33	19	31.67

A paired‐samples *t*‐test was conducted to compare the scores of the two tests for the controlled experiment. Three other paired‐samples *t*‐tests were conducted to compare the test scores, eye movements, and reading speeds under conditions before and after exercise for the variable experiment. Table 2 summarizes the categories that were compared in the paired‐samples *t*‐tests. The results of the paired‐samples *t*‐test are shown in Table 3.

**TABLE 2:** Paired‐samples *t*‐test categories

Category	Broad categories	Specific categories	
		First test	Second test
1	Scores in controlled experiment	Number of correctly scored questions	Number of correctly scored questions
2	Scores in variable experiment	Number of correctly scored questions before exercise	Number of correctly scored questions after exercise
3	Eye drifts in variable experiment	Number of time eye drifts away from laptop screen before exercise	Number of time eye drifts away from laptop screen after exercise
4	Reading speeds in variable experiment	Words per second read before exercise	Words per second read after exercise

**TABLE 3:** Paired‐samples *T*‐test results for scores and attention indices

First versus second test: Categories	Mean difference	SD difference	SE mean difference	95% confidence interval of the difference		t	df	One‐sided p	Two‐sided p
				Lower	Upper				
1	0.20000	1.12648	0.20567	−0.22064	0.62064	0.972	29	.169	.339
2	−1.26667	0.90719	0.16563	−1.60542	−0.92792	−7.648	29	<.001	<.001
3	1.33333	1.49328	0.27263	0.77573	1.89093	4.891	29	<.001	<.001
4	−1.03567	0.88921	0.16235	−1.36770	−0.70363	−6.379	29	<.001	<.001

The samples are continuous, approximately normally distributed, and do not contain outliers. The samples also passed Levene's test for homogeneity of variance. For the controlled experiment, the subjects only averaged 0.20000 points more in the first test. The two‐tailed *p*‐value was .169, which is less than .05. Thus, there was not a significant difference in the scores for the two tests.

In the variable experiment with exercise, the mean score before exercise was 7.1667, while the mean score after exercise was 8.4333. There was a significant difference in the scores for the variable group. Further analysis of the data shows that there was a significant difference in the number of times the eye movement drifts out of the screen frame during the test before exercise, which had a mean of 2.6333, and the test after exercise, which had a mean of 1.3000. There was a significant difference in the reading speeds for the test before exercise, which had a mean of 3.33556 words per second, and the test after exercise, which had a mean of 4.3913 words per second. As shown in Table 3, the one‐tailed *p*‐values of the categories of the variable experiment were all less than .001, indicating significant differences. What follows is the analysis and interpretation of the mean differences and *p*‐values in the paired‐samples *t*‐tests.

## 5 DISCUSSION

### 5.1 Data analysis

Since there was not a significant difference between the two test scores of the controlled experiment, it could be inferred that there was no significant difference between the difficulty of the two tests. This shows that two tests were viable in testing the difference in scores and attention indices before and after exercise in the experimental group.

In the experimental experiment, there was a significantly higher number of times participants’ eye movements drift beyond the frame of the screen during the test before exercise. This suggests that during the test, participants’ attention was more susceptible to influence and distraction beyond the passage displayed on‐screen; after exercise, participants were more concentrated on their task. This result clearly aligns with the existing literature and claims of Budde et al. (2008), de Sousa et al. (2008), and Spitzer and Hollmann (2013) that physical exercise enhances subjects’ attention through activation in the prefrontal cortex and cerebellum.

In addition, the reading speed of the test taken after exercise was significantly greater than that of the test before the exercise, suggesting that exercise caused participants to more efficiently read and absorb information. Since it has been established in this study that physical exercise causes decreased eye drifts, a sign of inattention, the accompanying increase in reading speed could indicate and be connected to an increase of attention. This qualifies Miller's (2015) claim that longer reading times indicate enhanced attention only in self‐paced reading without limited time.

The results of these two attention indices suggest that participants were more attentive and engaged in test taking after exercise. The enhancement of attention could be reflected in the two scores of the experimental group. The test after exercise had higher scores than that before exercise. This affirms with Sievertsen et al. (2016) and McConaughy et al. (2009) that attention is an integral factor in standardized testing performance. Taken together, exercise caused participants to become more attentive in their tasks, which was accompanied by an increase in test scores. It could be logically confirmed that, in line with the literature, acute, coordinative physical exercises improve standardized test scores through enhanced attention, which was quantitatively measured and correlated by reading speed and the number of eye drifts. This study further consolidates that reading speed and eye movements could be used to correlate physical exercise and testing performance, as well as confirm with past studies that the indices are potential methods to quantify attention. Thus, these results validate the line of reasoning established in the literature, and the hypothesis was proven to be correct.

### 5.2 Implications

Since the hypothesis was proven correct, students could learn to better perform in standardized tests. While it should be noted that physical exercise does not necessarily increase students’ maximum testing performance and scores, it has the potential to help students concentrate and engage more attentively with the test to increase their performance closer to their maximum potential. This method could help decrease the fluctuation in an individual student's performance by decreasing the effects of external distractions (Jonikaitis et al., 2013). Thus, it could help schools more accurately and reliably assess students’ academic performance and aptitude. Students could replicate steps in the exercise experiment, without measuring attention, to evaluate if physical exercise helps improve standardized testing, before official tests. Schools could make it optional for students to access nearby sporting facilities before the test. With the recent announcement from the College Board to digitalize the SAT, this study provides further insight into future test‐taking strategies. Since standardized tests were done digitally in this experiment, the method could more realistically simulate that of an actual SAT test, making the results of this study more predictable in the future. Furthermore, this study verified the applicability of the two indices, reading time and eye drifts, in measuring attention.

### 5.3 Limitations

However, there are limitations. While the study did use eye movement to gauge and track attention, the study neglected the effect of head movement, one of the indices of attention per Nguyen et al. (2018). The rotation of the face by head movement makes it more difficult for dlib to create a facial landmark and OpenCV to track the pupil movement. If participants’ head position did fluctuate throughout the test, there may be inaccuracies in the detected number of times the eye drifts, thus affecting the measured degree of attention (Wu & Ji, 2018). If the degree of head rotation was large enough to create inaccuracies in eye movement tracking, it could be inferred that the participant's attention was not focused on the screen, making it a potential factor in the experiment.

Another limitation is the difference in required exercise recovery time for each person. While age and heart rates have been accounted for in exercise intensity for each subject, the recovery time was standardized to 5 min as suggested by Cantrelle et al. (2020) for all subjects. However, resting time could potentially affect participants’ level of attention (Gutmann et al., 2018). Too much resting time could cause the effect of the acute exercise to lessen, but too little resting time could create excessive fatigue for participants, leading to less focus on the tests.

### 5.4 Future directions

To address a limitation, future research could include head movement as an index in measuring attention. Similar to how eye movement measures attention, the degree of rotation of the participants’ face could be indicative of where their attention is directed toward. Accounting head movement could also make eye movement more accurate, as specified in the limitations. In addition, future works could evaluate the effectiveness of various attention indices in predicting testing performance. Since this study uses running as an acute, coordinative, aerobic exercise, future studies could explore the effects of other acute, coordinative, aerobic exercises or other physical exercises with different intensities, such as walking as suggested by Hillman et al. (2009), on attention and standardized testing scores. To investigate the applicability of this study in real life, natural experiments could be conducted to compare attention and standardized test results of a class of students right before a physical exercise course and another one right after. Not only does the sample increase, but the results of the natural experiment could also provide suggestions to school curriculums to maximize student performance in standardized testing. Future research could develop on this study and better simulate the standardized tests in the future. Digital tests with formats and lengths more similar to those of the official SATs could be used to better simulate the conditions of a real test. Moreover, since this experiment only used the SAT exam as the standardized test, future research could replicate this experiment with different standardized tests and examine the effect of physical exercise on them.

## 6 CONCLUSION

This study affirmed the correlation between physical exercise and standardized test scores, as outlined in the literature, by evaluating and quantifying attention. It demonstrated that acute, coordinative, aerobic exercise could improve subjects’ attention, as suggested by decreased eye drifts and increased reading speed. The improvement of attention was then reflected in the increased standardized test scores after the physical exercise. This study validates the line of reasoning of past works and suggests physical exercise as a potential means to help students maximize their standardized testing performances. Future research could focus on including head movement as an attention index, replicate the experiment on different standardized tests or exercises, and conduct natural experiments to better simulate real‐life conditions to increase applicability.

### PEER REVIEW

The peer review history for this article is available at https://publons.com/publon/10.1002/brb3.2800

## Data Availability

Data used to support the findings of the study are within the article.

## References

[brb32800-bib-0001] Aslan, S. , Cataltepe, Z. , Diner, I. , Dundar, O. , Esme, A. A. , Ferens, R. , Kamhi, G. , Oktay, E. , Soysal, C. , & Yener, M. (2014). Learner engagement measurement and classification in 1:1 learning . 2014 13th International Conference on Machine Learning and Applications. 10.1109/icmla.2014.111

[brb32800-bib-0002] Azevedo, R. (2015). Defining and measuring engagement and learning in science: Conceptual, theoretical, methodological, and analytical issues. Educational Psychologist, 50(1), 84–94. 10.1080/00461520.2015.1004069

[brb32800-bib-0003] Bates, M. E. , & Lemay, E. P. (2004). The D2 test of attention: Construct validity and extensions in scoring techniques. Journal of the International Neuropsychological Society, 10(03), 392–400. 10.1017/s135561770410307x 15147597

[brb32800-bib-0004] Benjamin, A. S. , & Pashler, H. (2015). The value of standardized testing. Policy Insights from the Behavioral and Brain Sciences, 2(1), 13–23. 10.1177/2372732215601116

[brb32800-bib-0005] Boyko, N. , Basystiuk, O. , & Shakhovska, N. (2018). Performance evaluation and comparison of software for face recognition, based on dlib and OpenCV library . 2018 IEEE Second International Conference on Data Stream Mining & Processing (DSMP). 10.1109/dsmp.2018.8478556

[brb32800-bib-0006] Brickenkamp, R. (1962). Test d2: Aufmerksamkeits‐belastungs‐test. Hogrefe.

[brb32800-bib-0007] Budde, H. , Voelcker‐Rehage, C. , Pietraßyk‐Kendziorra, S. , Ribeiro, P. , & Tidow, G. (2008). Acute coordinative exercise improves attentional performance in adolescents. Neuroscience Letters, 441(2), 219–223. 10.1016/j.neulet.2008.06.024 18602754

[brb32800-bib-0008] Cantrelle, J. , Burnett, G. , & Loprinzi, P. D. (2020). Acute exercise on memory function: Open vs. closed skilled exercise. Health Promotion Perspectives, 10(2), 123–128. 10.34172/hpp.2020.20 32296624 PMC7146046

[brb32800-bib-0009] College Board . (2020). Chapter 5 about the SAT reading test . https://satsuite.collegeboard.org/media/pdf/official‐sat‐study‐guide‐about‐reading‐test.pdf

[brb32800-bib-0010] College Board . (2021). The Digital Sat Suite of assessments . https://satsuite.collegeboard.org/digital

[brb32800-bib-0011] College Board . (2022). The reading test: Overview . https://satsuite.collegeboard.org/sat/whats‐on‐the‐test/reading/overview

[brb32800-bib-0012] Choi, J. W. , Han, D. H. , Kang, K. D. , Jung, H. Y. , & Renshaw, P. F. (2015). Aerobic exercise and attention deficit hyperactivity disorder. Medicine & Science in Sports & Exercise, 47(1), 33–39. 10.1249/mss.0000000000000373 24824770 PMC5504911

[brb32800-bib-0013] Coe, D. P. , Pivarnik, J. M. , Womack, C. J. , Reeves, M. J. , & Malina, R. M. (2006). Effect of physical education and activity levels on academic achievement in children. Medicine & Science in Sports & Exercise, 38(8), 1515–1519. 10.1249/01.mss.0000227537.13175.1b 16888468

[brb32800-bib-0014] Coltheart, M. (1987). Attention and performance XII: The psychology of reading. Lawrence Erlbaum Associates.

[brb32800-bib-0015] de Sousa, F. M. A. , Medeiros, A. R. , Del Rosso, S. , Stults‐Kolehmainen, M. , & Boullosa, D. A. (2018). The influence of exercise and physical fitness status on attention: A systematic review. International Review of Sport and Exercise Psychology, 12(1), 202–234. 10.1080/1750984x.2018.1455889

[brb32800-bib-0016] Diamond, A. (2011). Biological and social influences on cognitive control processes dependent on prefrontal cortex. Progress in Brain Research, 189, 319–339. 10.1016/b978-0-444-53884-0.00032-4 21489397 PMC4103914

[brb32800-bib-0017] Duchowski, A. (2017). Eye tracking methodology: Theory and practice. Springer.

[brb32800-bib-0018] Ellis, J. J. , Glaholt, M. G. , & Reingold, E. M. (2011). Eye movements reveal solution knowledge prior to insight. Consciousness and Cognition, 20(3), 768–776. 10.1016/j.concog.2010.12.007 21273095

[brb32800-bib-0019] Gutmann, B. , Zimmer, P. , Hülsdünker, T. , Lefebvre, J. , Binnebößel, S. , Oberste, M. , Bloch, W. , Strüder, H. K. , & Mierau, A. (2018). The effects of exercise intensity and post‐exercise recovery time on cortical activation as revealed by EEG Alpha Peak Frequency. Neuroscience Letters, 668, 159–163. 10.1016/j.neulet.2018.01.007 29329910

[brb32800-bib-0020] Hart, R. , Casserly, M. , Uzzell, R. , Palacios, M. , Corcoran, A. , & Spurgeon, L. (2015). Student testing in America's great city schools: An inventory and preliminary analysis. Council of the Great City Schools.

[brb32800-bib-0021] Hillman, C. , Pontifex, M. , Raine, L. , Castelli, D. , Hall, E. , & Kramer, A. (2009). The effect of acute treadmill walking on cognitive control and academic achievement in preadolescent children. Neuroscience, 159(3), 1044–1054. 10.1016/j.neuroscience.2009.01.057 19356688 PMC2667807

[brb32800-bib-0022] Himelfarb, I. (2019). A primer on standardized testing: History, measurement, classical test theory, item response theory, and equating. Journal of Chiropractic Education, 33(2), 151–163. 10.7899/jce-18-22 31169998 PMC6759012

[brb32800-bib-0023] Jonikaitis, D. , Szinte, M. , Rolfs, M. , & Cavanagh, P. (2013). Allocation of attention across saccades. Journal of Neurophysiology, 109(5), 1425–1434. 10.1152/jn.00656.2012 23221410

[brb32800-bib-0024] Karvonen, J. , & Vuorimaa, T. (1988). Heart rate and exercise intensity during sports activities. Sports Medicine, 5(5), 303–312. 10.2165/00007256-198805050-00002 3387734

[brb32800-bib-0025] Katz, L. A. , & Carlisle, J. F. (2009). Teaching students with reading difficulties to be close readers: A feasibility study. Language, Speech, and Hearing Services in Schools, 40(3), 325–340. 10.1044/0161-1461(2009/07-0096) 19564445

[brb32800-bib-0026] Kazemi, V. , & Sullivan, J. (2014). One millisecond face alignment with an ensemble of regression trees . 2014 IEEE Conference on Computer Vision and Pattern Recognition. 10.1109/cvpr.2014.241

[brb32800-bib-0027] Kempf, A. (2016). The pedagogy of standardized testing: The radical impacts of educational standardization in the US and Canada. Palgrave Macmillan. 10.1057/9781137486653

[brb32800-bib-0028] Koegel, L. K. , Koegel, R. L. , & Smith, A. (1997). Variables related to differences in standardized test outcomes for children with autism. Journal of Autism and Developmental Disorders, 27(3), 233–243. 10.1023/a:1025894213424 9229256

[brb32800-bib-0029] Kwak, S. G. , & Kim, J. H. (2017). Central limit theorem: The cornerstone of modern statistics. Korean Journal of Anesthesiology, 70(2), 144–156. 10.4097/kjae.2017.70.2.144 28367284 PMC5370305

[brb32800-bib-0030] Leelavathy, S. , Jaichandran, R. , Shantha, S. K. , Surendar, B. , Aswin, K. P. , & Dekka, R. R. (2020). Students attention and engagement prediction using machine learning techniques. European Journal of Molecular & Clinical Medicine, 7(4), 3011–3017.

[brb32800-bib-0031] Liguori, G. , Feito, Y. , Fountaine, C. , & Roy, B. A. (2022). ACSM's guidelines for exercise testing and prescription. Wolters Kluwer.

[brb32800-bib-0032] Mandelbrot, B. (1961). Contributions to probability and statistics: Essays in honor of Harold Hotelling (Ingram Olkin, Sudhist G. Ghurye, Wassily Hoeffding, William G. Madow, and Henry B. Mann, eds.). SIAM Review, 3(1), 80–80. 10.1137/1003016

[brb32800-bib-0033] McConaughy, S. H. , Ivanova, M. Y. , Antshel, K. , & Eiraldi, R. B. (2009). Standardized observational assessment of attention deficit hyperactivity disorder combined and predominantly inattentive subtypes. I. Test session observations. School Psychology Review, 38(1), 45–66. 10.1080/02796015.2009.12087849 20802814 PMC2929017

[brb32800-bib-0034] Miller, B. W. (2015). Using reading times and eye‐movements to measure cognitive engagement. Educational Psychologist, 50(1), 31–42. 10.1080/00461520.2015.1004068

[brb32800-bib-0035] Morimoto, C. H. , & Mimica, M. R. (2005). Eye gaze tracking techniques for interactive applications. Computer Vision and Image Understanding, 98(1), 4–24. 10.1016/j.cviu.2004.07.010

[brb32800-bib-0036] Nguyen, A. , Yan, Z. , & Nahrstedt, K. (2018). Your attention is unique . Proceedings of the 26th ACM International Conference on Multimedia. 10.1145/3240508.3240669

[brb32800-bib-0037] Planinsec, J. (2002). Relations between the motor and cognitive dimensions of preschool girls and boys. Perceptual and Motor Skills, 94(2), 415–423. 10.2466/pms.2002.94.2.415 12027332

[brb32800-bib-0038] Reddy, G. P. (2018). Digital image processing: Principles and applications. In G. Reddy , & S. Singh (Eds.), Geotechnologies and the environment (pp. 101–126). Springer. 10.1007/978-3-319-78711-4_6

[brb32800-bib-0039] Sacks, P. (2000). Standardized minds: The high price of America's testing culture and what we can do to change it. Da Capo Press.

[brb32800-bib-0040] Schindler, S. , & Bublatzky, F. (2020). Attention and emotion: An integrative review of emotional face processing as a function of attention. Cortex, 130, 362–386. 10.1016/j.cortex.2020.06.010 32745728

[brb32800-bib-0041] Schwarz, L. , Gamba, H. R. , Pacheco, F. C. , Ramos, R. B. , & Sovierzoski, M. A. (2012). Pupil and iris detection in dynamic pupillometry using the OpenCV library . 2012 5th International Congress on Image and Signal Processing. 10.1109/cisp.2012.6469846

[brb32800-bib-0042] Sedgwick, P. , & Greenwood, N. (2015). Understanding the Hawthorne effect. BMJ, 351, h4672. 10.1136/bmj.h4672 26341898

[brb32800-bib-0043] Shang, L. , Zhang, C. , & Wu, H. (2019). Eye focus detection based on OpenCV . 2019 6th International Conference on Systems and Informatics (ICSAI). 10.1109/icsai48974.2019.9010199

[brb32800-bib-0044] Siddiqui, N. I. , Nessa, A. , & Hossain, M. A. (2010). Regular physical exercise: Way to healthy. Mymensingh Medical Journal, 19(1), 154–158. 20046192

[brb32800-bib-0045] Sievertsen, H. H. , Gino, F. , & Piovesan, M. (2016). Cognitive fatigue influences students’ performance on standardized tests. Proceedings of the National Academy of Sciences of the United States of America, 113(10), 2621–2624. 10.1073/pnas.1516947113 26884183 PMC4790980

[brb32800-bib-0046] Sinatra, G. M. , Heddy, B. C. , & Lombardi, D. (2015). The challenges of defining and measuring student engagement in science. Educational Psychologist, 50(1), 1–13. 10.1080/00461520.2014.1002924

[brb32800-bib-0047] Slutsky‐Ganesh, A. B. , Etnier, J. L. , & Labban, J. D. (2020). Acute exercise, memory, and neural activation in young adults. International Journal of Psychophysiology, 158, 299–309. 10.1016/j.ijpsycho.2020.09.018 33164850

[brb32800-bib-0048] Spitzer, U. S. , & Hollmann, W. (2013). Experimental observations of the effects of physical exercise on attention, academic and prosocial performance in school settings. Trends in Neuroscience and Education, 2(1), 1–6. 10.1016/j.tine.2013.03.002

[brb32800-bib-0049] Steinborn, M. B. , Langner, R. , Flehmig, H. C. , & Huestegge, L. (2018). Methodology of performance scoring in the D2 sustained‐attention test: Cumulative‐reliability functions and practical guidelines. Psychological Assessment, 30(3), 339–357. 10.1037/pas0000482 28406669

[brb32800-bib-0050] Student . (1908). The probable error of a mean. Biometrika, 6(1), 1–25. 10.2307/2331554

[brb32800-bib-0051] Viola, P. , & Jones, M. (2001). Rapid object detection using a boosted cascade of simple features . Proceedings of the 2001 IEEE Computer Society Conference on Computer Vision and Pattern Recognition. CVPR 2001. 10.1109/cvpr.2001.990517

[brb32800-bib-0052] Wang, C. , Chu, C. , Chu, I. , Chan, K. , & Chang, Y. (2013). Executive function during acute exercise: The role of exercise intensity. Journal of Sport and Exercise Psychology, 35(4), 358–367. 10.1123/jsep.35.4.358 23966446

[brb32800-bib-0053] Wiliam, D. (2010). Standardized testing and school accountability. Educational Psychologist, 45(2), 107–122. 10.1080/00461521003703060

[brb32800-bib-0054] Wingate, T. G. , & Tomes, J. L. (2017). Who's getting the grades and who's keeping them? A person‐centered approach to academic performance and performance variability. Learning and Individual Differences, 56, 175–182. 10.1016/j.lindif.2017.02.007

[brb32800-bib-0055] Wu, Y. , & Ji, Q. (2018). Facial landmark detection: A literature survey. International Journal of Computer Vision, 127(2), 115–142. 10.1007/s11263-018-1097-z

[brb32800-bib-0056] Zhao, M. , Gersch, T. M. , Schnitzer, B. S. , Dosher, B. A. , & Kowler, E. (2012). Eye movements and attention: The role of pre‐saccadic shifts of attention in perception, memory and the control of saccades. Vision Research, 74, 40–60. 10.1016/j.visres.2012.06.017 22809798 PMC3623695

[brb32800-bib-0057] Zwick, R. (2004). Rethinking the SAT: The future of standardized testing in university admissions. Taylor and Francis. 10.4324/9780203463932

[brb32800-bib-0058] Zwick, R. (2017). Who gets in? ‐ Strategies for fair and effective college admissions. Harvard University Press.

